# Transdisciplinary Innovations in Athlete Health: 3D-Printable Wearable Sensors for Health Monitoring and Sports Psychology

**DOI:** 10.3390/s25051453

**Published:** 2025-02-27

**Authors:** Mustafa Onder Sekeroglu, Metin Pekgor, Aydolu Algin, Turhan Toros, Emre Serin, Meliha Uzun, Gunay Cerit, Tugba Onat, Sermin Agrali Ermis

**Affiliations:** 1 Faculty of Sport Sciences, Mus Alparslan University, Mus 49001, Türkiye; mo.sekeroglu@alparslan.edu.tr; 2 Department of Mechanical and Product Design Engineering, Swinburne University of Technology, Melbourne, VIC 3122, Australia; metin.pekgor@outlook.com; 3 Faculty of Medicine, Akdeniz University, Antalya 07058, Türkiye; 4 Department of Coaching Education, Faculty of Sport Sciences, Mersin University, Mersin 33110, Türkiye; turhantoros@yahoo.com (T.T.); emreserin@mersin.edu.tr (E.S.); 5 High School of Physical Education and Sports, Sirnak University, Sirnak 73000, Türkiye; melihauzunn16@gmail.com (M.U.); gunaycerit60@gmail.com (G.C.); 6 Faculty of Sport Sciences, Hakkari University, Hakkari 30000, Türkiye; tugbaonat@hakkari.edu.tr; 7 Faculty of Sport Sciences, Aydin Adnan Menderes University, Aydin 09010, Türkiye; sermin.agrali@gmail.com

**Keywords:** sports psychology, wearable sensors, 3D printing, health monitoring, athlete performance

## Abstract

The integration of 3D printing technology into wearable sensor systems has catalyzed a paradigm shift in sports psychology and athlete health monitoring by enabling real-time, personalized data collection on physiological and psychological states. In this study, not only is the technical potential of these advancements examined but their real-world applications in sports psychology are also critically assessed. While the existing research primarily focuses on sensor fabrication and data acquisition, a significant gap remains in the evaluation of their direct impact on decision-making processes in coaching, mental resilience, and long-term psychological adaptation in athletes. A critical analysis of the current state of 3D-printed wearable sensors is conducted, highlighting both their advantages and limitations. By combining theoretical insights with practical considerations, a comprehensive framework is established for understanding how sensor-based interventions can be effectively incorporated into sports training and psychological evaluation. Future research should prioritize longitudinal studies, athlete-centered validation, and interdisciplinary collaborations to bridge the gap between technological developments and real-world applications. Additionally, the integration of artificial intelligence and advanced biomaterials has significant potential to enhance the reliability and interpretability of sensor-driven interventions. However, without rigorous scientific validation, their effectiveness remains uncertain. This study highlights the importance of a systematic approach in implementing and evaluating 3D-printed wearable sensors in sports psychology.

## 1. Introduction

Health is defined as a state of complete physical, mental, and social well-being, essential for optimal human functioning [1]. This definition underscores an individual’s capacity to adapt and manage challenges, a crucial aspect in sports where physical and psychological stresses are interconnected. Physical challenges in sports can lead to psychological consequences, manifesting as cognitive, emotional, and behavioral issues, while psychological challenges like relational problems, traumatic stress, anxiety, depression, aggression, disordered eating, and substance use can lead to serious physical consequences [2]. These interconnected stresses can impact an athlete’s performance, training, career transitions, interpersonal functioning, and physical rehabilitation unless they are properly addressed.

Sports psychology, the study of how psychological factors influence athletic performance, physical activity, and sports participation, integrates psychology, kinesiology, and physiology to optimize athletes’ mental well-being and performance. Understanding the complex relationship between the mind and body is crucial for developing effective training programs, enhancing performance, and promoting overall mental health in sports [1,3,4].

To further this understanding, wearable sensors play a pivotal role. These sophisticated instruments are worn on different body regions to monitor and analyze real-time physiological, biochemical, and electrophysiological outputs [5,6,7,8,9,10,11,12,13]. The sensors are positioned on areas such as the knees, feet, or hips to evaluate aspects of human motion like gait, and are equipped with a range of sensor types, including gyroscopes, force sensors, accelerometers, and electromyography, to collect precise data on physical activity. They also monitor biochemical and electrical impulses within the body, tracking physiological characteristics such as pulse rate (PR), heart period variability (HPV), resting pulse rate (RPR), and breathing frequency (BF) [14,15,16].

These devices provide uninterrupted, non-intrusive surveillance crucial for the prevention, treatment, and control of illnesses as well as for the maintenance of general health. Moreover, wearable sensor systems enable athletes to self-monitor and manage their psychological and physiological states, thereby enhancing their self-awareness and performance control [17]. As these sensors become increasingly important tools in sports psychology, they play a crucial role in maximizing athlete care and outcomes. The data collected by these sensors can be easily transmitted to the cloud through the Internet of Things (IoT) and 5G technologies, allowing sports professionals to analyze athletes’ conditions and develop customized training or intervention programs [18]. With ongoing advancements in data processing technologies such as cloud computing, machine learning, and artificial intelligence, raw sensor data can be transformed into actionable insights to identify potential issues before they impact performance [17].

Drawing on examples from the literature, the application of wearable sensors in sports psychology facilitates an unprecedented level of detailed and real-time monitoring of key physiological markers such as heart rate variability (HRV) and cortisol levels [19,20]. This capability allows sports psychologists to conduct sophisticated real-time analyses of an athlete’s stress responses and recovery dynamics. By incorporating electrocardiogram (ECG) and electromyography (EMG) sensors into wearable devices, continuous data collection is made possible, laying the groundwork for developing tailored recovery programs [19] that specifically address the demands following rigorous training sessions or competitions. This ensures that athletes maintain optimal mental and physical health.

Moreover, wearable sensors provide immediate feedback on critical physiological functions including breathing rate, heart rate, and muscle tension [21]. This information is essential for biofeedback training, where athletes learn to modify their physiological responses to enhance relaxation and performance under pressure. Training athletes to control their physiological processes during high-stress situations leads to improved focus and performance during critical competition moments [22].

In the realm of injury rehabilitation, wearable sensors play a pivotal role [23]. They continuously monitor the biomechanics of an athlete’s movements, identifying deviations from normal patterns that might indicate an increased risk of injury or an incomplete recovery from a previous injury. The immediate feedback provided by these sensors enables prompt adjustments in therapy and training, which help prevent re-injury and ensure a safe return to sport.

On the other hand, 3D printing has revolutionized the development of wearable sensors by enabling the creation of highly customizable, flexible, and precisely engineered devices tailored to athletes’ needs. Three-dimensionally printable wearable sensors, utilizing advanced materials and manufacturing techniques, have demonstrated significant utility in sports settings. For instance, tribo-electric sensors leverage fused deposition modeling (FDM), a widely used 3D printing method, to fabricate flexible thermoplastic polyurethane (TPU)-based structures that conform seamlessly to different body parts [24]. Similarly, strain sensors benefit from multi-material 3D printing, allowing the integration of silicone and conductive polymers into a single, flexible structure, significantly enhancing sensitivity and adaptability [25]. Furthermore, graphene-based sensors employ laser-induced graphene (LIG) fabrication, a technique that incorporates 3D printing principles to create highly conductive and lightweight wearable components [26]. Piezoresistive sensors utilize direct ink writing (DIW), an advanced 3D printing process, to deposit a composite of milled carbon fiber and silicone with precise control over mechanical properties, ensuring superior responsiveness and durability [27].

The role of 3D printing in sports psychology applications is transformative, as it enables the production of ultra-lightweight, comfortable, and non-intrusive sensors that can be seamlessly integrated into sportswear or worn directly on the skin. Methods like FDM and DIW ensure that these sensors provide a perfect anatomical fit, eliminating discomfort and minimizing distractions during high-performance activities. Additionally, 3D-printed sensors provide real-time physiological and biomechanical data, such as muscle activity, joint movement, and force exertion, allowing sports psychologists to assess how athletes respond to both physical exertion and psychological stress in competitive environments. This data-driven approach, made possible by 3D printing technologies, is essential for designing personalized mental training programs aimed at improving psychological resilience, focus, and performance. Moreover, by continuously monitoring physiological responses across different phases of activity, sports psychologists can gain unprecedented insights into the interplay between mental states and physical performance, paving the way for more effective interventions in anxiety management and performance optimization.

**Figure 1:**
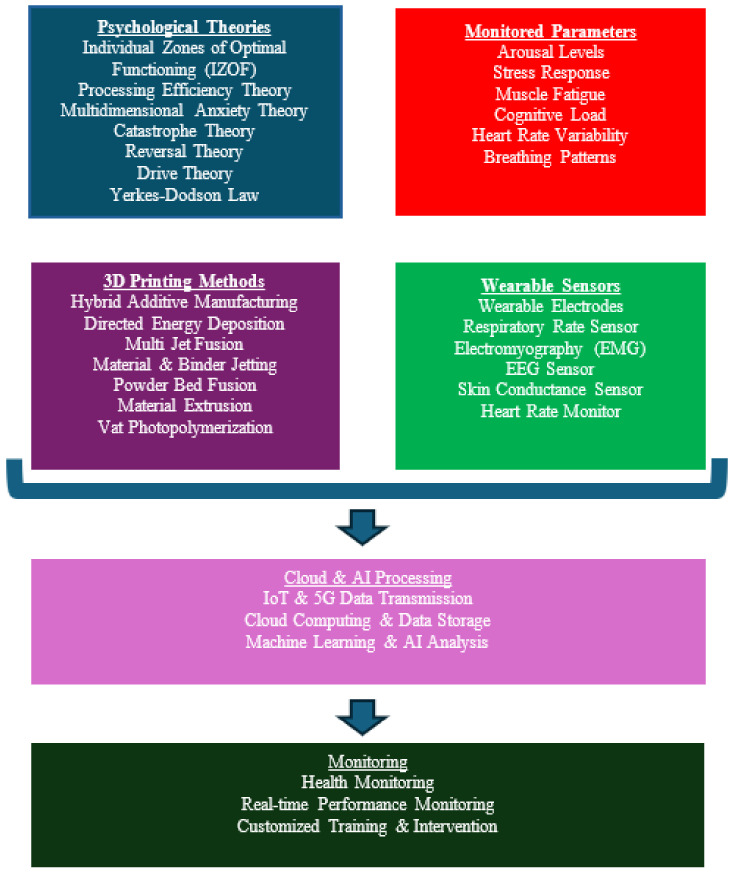
A multi-dimensional approach to athlete monitoring: sports psychology, wearable sensors, and advanced manufacturing.

In this study, a comprehensive framework was established to examine the intricate relationship between physical and psychological well-being in athletes. The discussion will now progress to more targeted applications of these insights. The following sections will explore the sophisticated realm of wearable sensors, highlighting their critical role not merely as monitoring devices but as catalysts for enhanced sports performance through real-time analytical capabilities. Various sports psychology theories that leverage these data to refine training and intervention strategies will be examined, ultimately enhancing both mental resilience and physical performance. Additionally, the innovative 3D printing technologies and the materials used for 3D-printed wearables, which are pivotal to the manufacturing of these advanced sensors, will be detailed, underscoring the fusion of cutting-edge technology with sports science. This integration marks a new era in sports psychology, where technological advancements and theoretical insights converge to foster a deeper understanding of and stronger support for athletes’ health and performance (Figure 1).

## 2. Sports Psychology and Wearable Sensors

Sports psychology delves into the intricate relationship between arousal, emotion, and performance, significantly enriched by the advent of wearable sensor technologies. These devices capably monitor physiological indicators such as heart rate, skin conductance, and muscle activity in real time, providing precise data crucial for understanding an athlete’s arousal and emotional states. These precise data facilitate the development of personalized training and intervention strategies, crucial for optimizing athletic performance [5,6,7,8,9,10,11,12,13].

**Figure 2:**
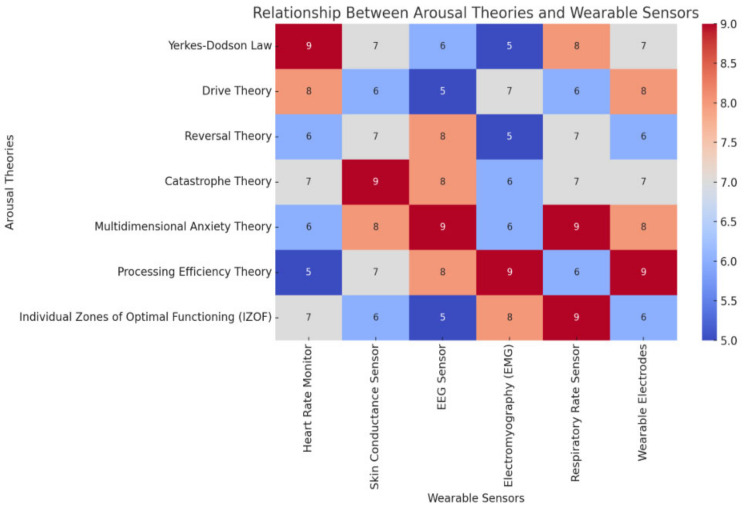
Mapping arousal theories to wearable sensor applications in sports psychology.

Theoretical frameworks in sports psychology provide insights into how various levels and types of arousal impact performance. For instance, the Yerkes–Dodson Law proposes an optimal arousal level for peak performance that varies with the task’s complexity [28]. Simple tasks may benefit from higher arousal, enhancing performance, whereas complex tasks typically require lower arousal levels. Drive Theory suggests a direct relationship between increased arousal and advanced performance, especially in well-learned tasks [29]. Conversely, Reversal Theory, developed by Michael Apter, interprets arousal subjectively, suggesting that athletes can shift their arousal states to elevate performance through cognitive strategies [30]. Catastrophe Theory, proposed by J.G. Hardy, highlights a nonlinear relationship between arousal and performance, emphasizing the need to manage physiological arousal and cognitive anxiety to prevent performance drops [31]. The Individual Zones of Optimal Functioning (IZOF) model posits that each athlete has a unique optimal arousal level that maximizes their performance, underscoring the importance of personalized arousal management [32]. These theories are visualized in Figure 2 and Table 1, which map the theories to their potential applications in sports psychology using wearable sensors.

Advanced wearable sensor technologies, including smart materials, nanotechnology, and 3D printing, allow sports psychologists and coaches to monitor and manage an athlete’s psychological status and arousal levels in real-time. This multidisciplinary approach facilitates personalized interventions and real-time feedback, significantly enhancing athletic performance and mental well-being.

The landscape of wearable devices and mobile applications for monitoring psychological stress, brain activity, and cognitive function is notably diverse and innovative. Notable applications include Opti Brain™ paired with the Muse™ headband for advanced electroencephalography (EEG) analysis and the T2 Mood Tracker, which monitors emotional health [38]. Devices like the King–Devick Test and HitCheck are crucial for assessing cognitive function, particularly after concussions, while BrainCheck Sport focuses on attention and memory [38]. These examples illustrate the broad range of wearable technologies that are available for personal, medical, and research purposes, as shown in Table 2, which lists wearable sensor applications in health monitoring and sports psychology.

One of the most transformative advances in this field has been the integration of 3D printing. Initially pivotal for prototype design, 3D printing now facilitates the production of complex sensor structures that would be challenging with traditional manufacturing techniques. This technology enables the customization of sensors to precisely fit different body parts or meet individual users’ specific needs, boosted by the use of conductive polymers and nanomaterials that optimize sensor sensitivity and performance. The development of multilayered structures allows for the integration of electronic components directly during the printing process, optimizing sensor performance and size.

The next sections will explore the specific 3D printing techniques that enable the creation of these sophisticated sensors, focusing on the adaptability and precision required by sports professionals. This discussion underscores the critical role of modern manufacturing processes in advancing sports psychology by integrating cutting-edge technology and theoretical insights.

## 3. Three-Dimensional Printing Techniques for Wearable Sensors Used in Health Monitoring and Sports Psychology

**Figure 3:**
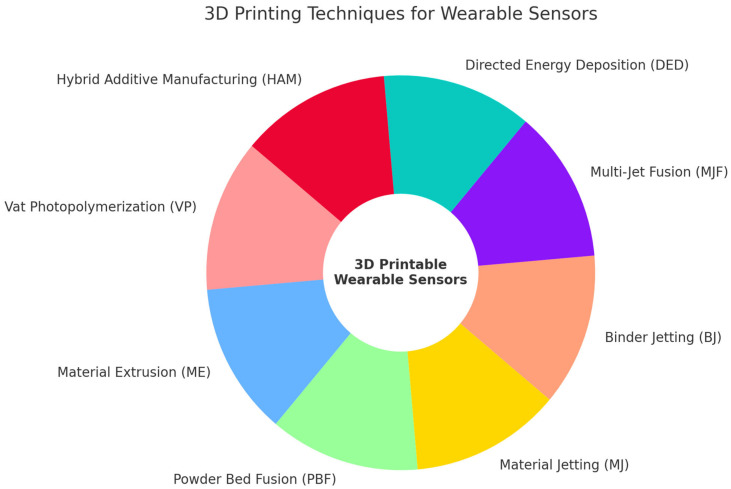
The fundamental methods utilized in 3D printing for wearable sensors.

3D printing, also known as additive manufacturing, is a versatile technology that encompasses a wide range of techniques, each suited to different materials including metals, polymers, and composites [39,40]. The procedures outlined in the ISO/ASTM 52900:2015 standards [39] allow for the layer-by-layer creation of products and offer the opportunity to select several types of materials during the printing process. In the printing process, first, a digital 3D model is created using a 3D scanner or computer-aided design (CAD) software. Then, the digital data are transferred to an STL file [40]. Afterwards, slicing software is utilized to convert the STL file into a G-code format [41]. The model is divided into a series of 2D levels, enabling the objects to be placed in a stacked manner, one layer at a time [42]. This digital process allows for the easy printing of various products, regardless of their geometry. Three-dimensional printing techniques can be utilized to explore multiple aspects of materials simultaneously, such as their electronic, fluidic, optical, acoustic, thermal, chemical, and electromagnetic properties, allowing for the creation of novel manufacturing procedures [39]. The fundamental methods utilized in 3D printing are elaborated below (Figure 3).

Wearable sensors fabricated using vat photopolymerization (VP) techniques have significantly broadened the scope of health monitoring applications. These sensors encompass a diverse range of functionalities, including glucose [43,44], lactate [43,44], sweat [45], and strain sensors [46,47,48], as well as tactile sensors and artificial skin [49], oximeters [50], smart bandages, and sensors designed for electroencephalography (EEG) and electrocardiography (ECG) [51]. The precision and customizability offered by VP allow for the creation of highly detailed and application-specific geometries, enhancing the functionality and effectiveness of these devices. Notably, while much of the current literature, including [51], focuses on the use of VP in wearable sensors, other studies have explored its application in broader contexts such as for optical components [52,53] and medical devices [54]. This breadth underscores the versatility of VP techniques, demonstrating their efficacy in producing not only wearable sensors but also a wide range of intricate devices across various fields. In summary, vat photopolymerization has been instrumental in producing an extensive array of wearable sensors for diverse healthcare monitoring purposes. These sensors utilize the high resolution and design flexibility of VP to meet the exact needs of wearable healthcare applications, showcasing the significant impact of this technology on the advancement of wearable devices [51].

Material extrusion (ME), notably through the popular techniques of fused deposition modeling (FDM) or fused filament fabrication (FFF), represents a cornerstone in 3D printing technology. In this method, thermoplastic filaments are heated and pushed through a nozzle to construct objects layer by layer [40]. This approach is extensively utilized across various domains such as prototyping, education, and the production of functional parts using a wide array of thermoplastics. The FDM process encompasses several variations like Precise Extrusion Deposition (PED), Precise Extrusion Manufacturing (PEM), and Multiphase Jet Solidification (MJS), which all involve melting materials during the printing process. Alternatively, there are non-melting processes such as solvent-based extrusion-free forming, Low-Temperature Deposition Manufacturing (LDM), Pressure-Assisted Microsyringe (PAM), and direct ink writing (DIW) [51]. Material extrusion (ME) has been instrumental in advancing the development of wearable sensors, enabling the precise monitoring of diverse parameters such as strain [55,56], glucose [57], lactate [58], and deformation [51]. This technique has been enriched by the introduction of conductive inks composed of soft thermoplastic elastomers mixed with conductive additives like silver micro flakes, carbon black nanoparticles, or poly(3,4-ethylenedioxythiophene) (PEDOT). These innovative inks are crucial for sensors that monitor strain, temperature, and electrocardiogram signals, showcasing significant strides in sensor functionality. Further advancements in this field include the development of core–shell filaments that merge a thermoplastic elastomer shell with an acrylonitrile butadiene styrene core, improving both the printability and flexibility of materials for cutting-edge wearable sensor applications [59,60]. Research has also been conducted on silicone elastomer sheets specifically designed for wearable biomedical devices, demonstrating the expansive application potential of ME even when its direct use is not explicitly detailed [61]. Additional innovations feature a multi-material micro-extrusion technique that facilitates direct deposition onto stretchable fabrics, significantly advancing the integration of functionality without compromising resolution [62]. The precision of filament-based techniques has been validated through the production of complex geometrical forms with high accuracy [60]. Furthermore, the meticulous characterization of the mechanical properties of silicone elastomer sheets through biaxial tensile testing confirms the precise control attainable over material properties with ME [63]. These examples underscore the versatility of material extrusion in meeting various functional requirements and its capacity to drive innovation in wearable technology. Collectively, these advancements highlight the ongoing evolution of 3D printing techniques in enhancing both the design and functionality of wearable devices, paving the way for their seamless integration into daily health monitoring and medical diagnostics.

Powder bed fusion (PBF) is a sophisticated 3D printing technique that encompasses both Selective Laser Sintering (SLS) and Electron Beam Melting (EBM). SLS employs a laser to sinter powdered materials such as plastics or metals, building objects layer by layer [64]. In contrast, EBM utilizes an electron beam in a vacuum to melt metal powder precisely, making it particularly suitable for crafting durable and complex structures commonly used in the aerospace and medical industries [65]. In the domain of wearable sensor technology, PBF is celebrated for its ability to fabricate components with exceptional strength and precision. An illustrative application of this technology includes the development of force sensors that incorporate a deformation element and a steel plate as a measuring element carrier. These sensors demonstrate PBF’s capability to produce devices with minimal linearity and hysteresis errors, underscoring the technology’s precision and reliability—essential qualities for sensors that must perform consistently under various conditions [66]. While specific applications of PBF in wearable sensors are well documented, the broader literature often focuses more on the technology’s capabilities than on detailed applications of wearable sensors. The materials typically used, featuring combinations such as a base body with a steel plate, exemplify PBF’s versatility and adeptness in handling complex material compositions. These traits are crucial for achieving high resolution and accuracy in the final products [67]. However, the prevalence of wearable sensors utilizing PBF is noted to be limited, indicating a fertile area for further exploration and documentation within the industry. This observation points to the potential for the expanded application of PBF in wearable technology, advocating for more extensive research and development to fully harness this technology’s capabilities [68].

Material and binder jetting are innovative 3D printing techniques that closely resemble the process of inkjet printing. Material jetting (MJ) involves the precise deposition of droplets of materials such as photopolymers or waxes, which are layered and then cured to form the final product [51]. Binder jetting (BJ), conversely, employs a liquid binding agent that is sprayed onto a powder bed, effectively bonding the material layers together [51]. These methods are especially valuable for producing full-color prototypes, intricate geometries, and multi-material components [65,66]. Extensive research into these technologies has led to the development of a variety of wearable (bio)sensors that monitor health-related metrics such as glucose, lactate, and sweat levels, in addition to strain, tactile feedback, and oxygen levels through wearable oximeters. These sensors, crafted with healthcare applications in mind, are distinguished by their exceptional stretchability, flexibility, cost-effectiveness, ultra-thinness, and lightweight properties. The materials used in these sensors, including composite filaments, elastomers, functional inks, and hydrogels, are particularly well suited to the MJ and BJ processes, optimizing their functionality and application versatility [51]. While the specific details of the resolution and accuracy of these sensors are not elaborated upon in the sources, it is recognized that MJ and BJ can achieve high levels of precision. This precision is critical for wearable sensors, as it allows the production of devices with complex shapes and fine details essential for accurately capturing and monitoring physiological data [65,66]. In conclusion, the utilization of material jetting and binder jetting technologies has facilitated the development of a diverse range of wearable sensors designed to effectively monitor various physiological parameters. These technologies not only enable the creation of highly functional sensors but also ensure that the sensors are adaptable, comfortable, and precise, making them ideal for continuous health monitoring. This adaptability and precision ensure that these sensors meet the rigorous demands of modern healthcare applications, demonstrating the significant impact of these 3D printing techniques in the field of wearable technology [51,65,66].

Multi-Jet Fusion (MJF) is a sophisticated powder bed fusion technique developed by HP that utilizes inkjet printheads to distribute a fusing agent across a powder layer. This agent is then sintered using a heat source, enabling the rapid construction of parts characterized by intricate details and robust mechanical properties. Renowned for its speed, precision, and versatility, MJF is particularly suited for fabricating parts with complex geometries and high dimensional accuracy. In wearable sensor technology, MJF has demonstrated its potential through the creation of sensors using conductive graphene nanoplate–carbon nanotube (GC) ink. These sensors, designed for healthcare applications, leverage MJF’s layer-by-layer printing capability to optimize both mechanical properties and sensor sensitivity [67]. The technology’s high-resolution capabilities are especially critical for producing sensors that demand precise dimensional accuracy, as evidenced in studies highlighting its effectiveness in achieving functional designs [68]. Further advancements include the development of voxelated conductive elastomers, showcasing MJF’s ability to handle complex material compositions and adjust electrical conductivities within printed parts [69]. This adaptability plays a pivotal role in enabling functional diversity, allowing sensors to be tailored for varied healthcare monitoring scenarios. By combining detailed structural design with functional precision, MJF has emerged as a powerful tool for the development of advanced wearable sensors. These attributes underscore MJF’s value not only in manufacturing intricate and reliable sensor components but also in pushing the boundaries of wearable technology. Its ability to seamlessly integrate mechanical and functional properties highlights its transformative impact in the fields of healthcare monitoring and wearable devices.

Directed Energy Deposition (DED) is an advanced 3D printing technique that employs focused thermal energy—such as a laser, electron beam, or plasma arc—to melt materials, typically in powder or wire form. The melted material is deposited layer by layer, enabling the fabrication of new parts or the repair and refinement of existing high-value components. Known for its precision and versatility, DED is particularly valuable in industries that demand complex, high-precision tasks, such as those of aerospace, automotives, and healthcare. A notable application of DED technology is the creation of piezoelectric sensors, such as Pyzoflex^®^, which transform mechanical stress into electrical charge—an essential feature for dynamic monitoring applications. These sensors are typically made from ceramics or polymers with piezoelectric properties, although their precise composition remains unspecified in the available documentation [70]. This functionality makes Pyzoflex^®^ sensors well suited for applications requiring the detailed and accurate monitoring of dynamic processes. Research has explored the role of these sensors in monitoring powder flow during laser-assisted Directed Energy Deposition (L-DED) processes [68,70]. These studies highlight the sensors’ high resolution and accuracy, emphasizing their real-time precision in tracking powder flow. Such capabilities demonstrate the effectiveness of piezoelectric sensors in ensuring process reliability and precision. Furthermore, these findings underscore the adaptability of DED technology in integrating sensor functionality with high-tech manufacturing processes. In summary, DED’s ability to combine precision, adaptability, and functionality makes it a powerful tool for developing advanced sensors and components. By enabling real-time monitoring and enhancing the accuracy of dynamic processes, DED showcases its potential as a transformative technology across multiple industries.

Hybrid Additive Manufacturing (HAM) combines the capabilities of 3D printing with the precision of subtractive techniques such as CNC machining. This innovative approach boosts the detail and surface finish of parts while enabling the creation of complex components with exceptional accuracy. By merging additive and subtractive methods, HAM addresses limitations in traditional manufacturing techniques, offering new possibilities for fabricating advanced materials and devices. One notable application of HAM involves the development of high-performance conductive polymer composites (CPCs) specifically tailored for wearable sensors. These materials, characterized by their flexibility and seamless integration potential, are particularly suitable for strain sensors embedded in clothing. This advancement highlights the versatility of HAM in producing functional components that align with the demands of modern wearable technology [71]. Further contributing to the field, new conductive photo-resins have been developed for additive manufacturing to create flexible conductive composites. These materials are ideal for wearable sensors requiring both adaptability and durability, offering a balance between functionality and resilience [72]. The integration of these innovations into HAM underscores its pivotal role in expanding the functionality and application range of wearable sensors, particularly in health monitoring within sports psychology. As illustrated in Table 3, Hybrid Additive Manufacturing demonstrates the widespread adoption and effectiveness of 3D printing techniques in creating advanced health monitoring devices. These sensors seamlessly integrate into everyday apparel and equipment, bridging the gap between cutting-edge technology and practical, user-friendly solutions. This multidisciplinary approach highlights the transformative potential of HAM in wearable sensor technology, paving the way for new possibilities in continuous health monitoring and sports psychology applications. Table 3 shows the 3D printing techniques for health monitoring with wearable sensors in sports psychology.

## 4. Synthesis of Materials for 3D-Printable Wearable Sensors in Sports Psychology: Past, Present, and Future

Building on the discussion of 3D printing methods for wearable sensors, the synthesis of materials for these applications has evolved significantly to meet the specific needs of sports psychology. The transition from rigid, cumbersome devices to biocompatible, stretchable, and smart materials has made these sensors more effective for continuous mental and physical health monitoring in athletes [74,75].

In the early stages of wearable sensor development, materials were largely composed of rigid silicon-based and metallic components. While these materials provided high precision, their inflexible structure made them impractical for the real-time tracking of an athlete’s stress responses, cognitive load, and emotional states during high-intensity training or competitions. The discomfort and restricted movement caused by these early sensors prevented athletes from using them consistently, limiting their effectiveness in psychological assessments and performance monitoring [74].

A major breakthrough in the late 1990s and early 2000s came with the introduction of flexible polymeric materials such as polydimethylsiloxane (PDMS), polyimide (PI), and flexible printed circuit boards (FPCBs) [76]. These materials enabled the development of lightweight, skin-adherent sensors that could be worn comfortably for extended periods. This advancement made psychophysiological monitoring more reliable and natural, allowing coaches, trainers, and sports psychologists to gain deeper insights into how stress, anxiety, and focus fluctuations affect performance. This shift laid the foundation for the development of the modern wearable sensor technologies that are now widely used to support mental and emotional resilience training in athletes.

The introduction of 3D printing and nanotechnology has further enhanced the potential of wearable sensors by improving their precision, flexibility, and adaptability. These innovations have enabled the creation of smarter, more effective materials that can monitor an athlete’s psychological and physiological state in real time, providing valuable feedback to help manage stress, optimize focus, and improve mental clarity.

One of the most significant advancements was the use of conductive polymers, such as poly(3,4-ethylenedioxythiophene): polystyrene sulfonate (PEDOT:PSS) [77,78]. These materials enhance the accuracy and sensitivity of electroencephalography (EEG) and heart rate variability (HRV) sensors, making it easier to track brain activity and cognitive load during training. By analyzing these data, athletes and their coaches can identify patterns of mental fatigue and adjust their training strategies accordingly.

Another major breakthrough was the integration of nanocomposites like carbon nanotubes (CNTs), graphene, and MXenes [79], which offer the high-precision tracking of stress biomarkers such as cortisol levels and HRV fluctuations. These ultra-sensitive materials allow for more accurate and timely assessments of mental stress and emotional regulation, helping athletes manage psychological pressure more effectively.

**Figure 4:**
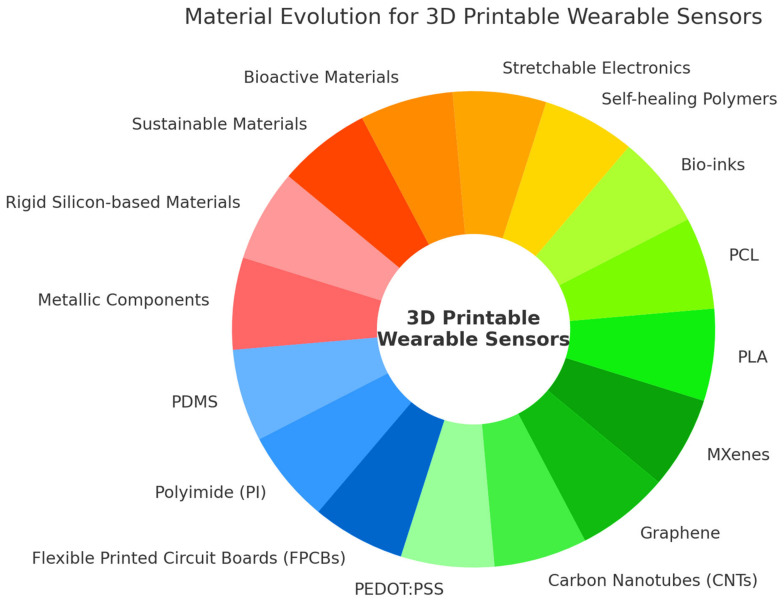
Materials for 3D-printable wearable sensors.

Additionally, biodegradable and biocompatible materials such as polylactic acid (PLA) [80], polycaprolactone (PCL) [81], and bio-inks [82] have been gaining increasing attention in sports technology and sports psychology, particularly in the development of wearable sensors for athlete monitoring. PLA, a widely used biodegradable polymer, is lightweight and adaptable, making it ideal for sensor-embedded sports gear such as smart textiles and adhesive patches. Its use in 3D-printed biosensors enhances data accuracy and sensor integration into sportswear without adding unnecessary bulk. On the other hand, PCL, with its slow degradation rate and high flexibility, is well suited for longer-term physiological tracking in endurance sports, where monitoring muscle fatigue, hydration levels, and stress responses can improve performance and injury prevention strategies. Furthermore, bio-inks have opened new possibilities for customized, athlete-specific wearable sensors that conform to individual physiological and psychological needs. These inks, composed of hydrogels, biomolecules, and living cells, are now being explored for next-generation bio-integrated sports devices that merge seamlessly with the human body. The adaptability of bio-inks ensures that sensors can be 3D-printed directly onto flexible materials or even onto the skin, allowing for the real-time monitoring of stress biomarkers such as cortisol fluctuations in competitive environments [82]. Figure 4 illustrates the materials used for 3D-printable wearable sensors.

Wearable sensors incorporating these materials allow for temporary yet effective psychophysiological monitoring without interfering with an athlete’s performance. Designed to be skin-friendly and flexible, these materials ensure that athletes can wear them comfortably during both training and competition without discomfort or movement restriction. This is particularly valuable in sports psychology, where the real-time tracking of stress levels, mental fatigue, and emotional states is crucial for developing personalized training regimens and psychological interventions.

As advancements in material science and sports psychology continue to intersect, the future of wearable sensors will emphasize the development of bio-adaptive, intelligent, and sustainable materials that can further enhance mental training and well-being. The next generation of wearable sensors will likely include the following:

- Self-healing and reconfigurable polymers [83], which will repair minor damage automatically. This feature will ensure long-term psychological and physiological monitoring without frequent sensor replacements. Such technology will be particularly beneficial in elite sports psychology, where athletes undergo intense mental and physical training over extended periods.
- Stretchable and shape-adaptive electronics [84], which will allow sensors to seamlessly conform to an athlete’s body movements and cognitive responses. These smart materials will enable real-time adjustments based on an athlete’s stress levels and mental state, providing adaptive focus training techniques that can be tailored to individual needs.
- Bioactive and smart materials [85,86] capable of detecting and analyzing stress hormones such as cortisol and lactate. These materials will allow for instant feedback on an athlete’s emotional and psychological state, making mental resilience training more data-driven and personalized.
- Sustainable and recyclable sensor materials [87], developed to support eco-friendly and ethical sports science practices. Materials such as lignin-based and cellulose-derived conductors will help reduce electronic waste, making wearable sensors for mental performance monitoring more sustainable while maintaining high precision and efficiency.

## 5. Future Directions and Innovations

The future of 3D-printable sensors in sports psychology is promising, with significant technological advancements in the field. In sports, sensor fusion often involves the integration of accelerometers, gyroscopes, and magnetometers. These technologies are applied in various sports, including athletics, swimming, cycling, and ball sports. Big data, paired with computer-based applications such as virtual reality (VR), augmented reality (AR), and neurotechnology, elevate the empirical and practical aspects of sports psychology, making it more appealing to athletes, multidisciplinary professionals, and investors. Sensor data collected pre- or post-3D printing can be utilized for digital twin applications, prototype development, and even mass production in remote areas. Artificial intelligence (AI) has the potential to transform sensor data analysis. Machine learning algorithms can identify patterns that may elude human analysts, leading to more sophisticated models for predicting performance and mental states. Advancements in material science will likely result in new materials that can further boost the sensor’s capabilities. Innovations in biocompatible and smart materials that respond dynamically to stimuli will expand the use of 3D-printable sensors in sports. The applications of 3D-printable sensors in sports psychology extend beyond performance and mental health monitoring. These sensors can be adapted for rehabilitation, helping monitor recovery in injured athletes. Their flexibility and customization potential make them ideal for tracking individualized recovery metrics and providing psychological support during recovery. Future research should encourage collaboration among engineers, psychologists, and sports scientists to optimize sensor design and application. This interdisciplinary approach will yield innovative solutions that advance both athletic performance and mental well-being.

## 6. Conclusions

Three-dimensional (3D) printing has revolutionized the design and production of wearable technology by enabling customized fits, self-sustaining wearables with built-in energy generation, and highly accurate sensors for health and environmental monitoring. Although it faces challenges, 3D printing remains essential for both prototyping and the large-scale production of wearable devices. This technology allows for the creation of configurable, lightweight devices that meet the practical demands for flexibility and low weight. However, significant hurdles remain, particularly in enhancing the mechanical and electrochemical properties of their components, such as batteries and supercapacitors [88]. Further research is required on the materials used in 3D printing, especially in developing printable organic, inorganic, and hybrid semiconductors, for the design of new sensors [89]. Additionally, challenges related to intellectual property and compliance with health regulations must be addressed, particularly in the creation of medical devices [90].

In conclusion, the integration of 3D printing technology with sports psychology is recognized as offering a unique opportunity to advance the understanding of athletic performance and well-being. By harnessing 3D-printable sensors, real-time data on physiological and psychological metrics can be collected by researchers and practitioners, leading to targeted interventions that can significantly improve athletic outcomes. As the field moves forward, it is crucial that the challenges related to data security, sensor accuracy, and acceptance among athletes and coaches be addressed. Advancements in AI and new materials are embraced, expanding the applications of 3D-printable sensors beyond performance monitoring to include health and wellness assessments.

By developing personalized interventions and employing cutting-edge technology, a more supportive and effective environment for athletes can be created, blending the best sports psychology practices with innovative technological advancements. This calls for collaboration among all stakeholders in the field to explore the untapped potential of 3D-printable sensors in sports psychology, ultimately fostering the generation of mentally resilient and physically adept athletes.

## Figures and Tables

**Table 1:** Some theories and applications in sports psychology.

Theory	Proponent(s)	Key Concept	Main Implications	Application in Sports Psychology	Possible Type of Sensors
Yerkes–Dodson Law [28]	Robert M. Yerkes, John D. Dodson	Optimal arousal level varies with task complexity.	Athletes need to find their optimal arousal level.	Helps determine the ideal arousal for different tasks.	Heart Rate Monitors
Drive Theory [29]	Clark Hull	Increased arousal boosts performance of well-learned tasks.	A linear relationship between arousal and performance.	Useful for tasks where high arousal is beneficial.	Electrocardiogram (ECG) Sensors
Reversal Theory [30]	Michael Apter	Arousal is subjectively interpreted as either pleasant or unpleasant.	Cognitive strategies can shift arousal perception.	Helps athletes manage and reinterpret their arousal.	Skin Conductance Sensors
Catastrophe Theory [31]	J.G. Hardy	Performance drops when arousal and cognitive anxiety exceed optimal levels.	Manages both arousal and anxiety to avoid performance drops.	Monitors combined physiological and psychological states.	Multi-Sensor Systems
Multidimensional Anxiety Theory [32]	Rainer Martens	Differentiates between cognitive and somatic anxiety.	Addresses both types of anxiety for optimal performance.	Helps tailor interventions for cognitive and somatic anxiety.	Wearable Electrodes
Processing Efficiency Theory [33]	Michael Eysenck, Manuel Calvo	Anxiety affects cognitive processes and task performance.	Reduces cognitive load and stress to maintain performance.	Monitors cognitive and stress levels for performance optimization.	Brain–Computer Interfaces (BCIs)
Individual Zones of Optimal Functioning (IZOF) [34]	Yuri Hanin	Each athlete has a unique optimal arousal level.	Personalized arousal management approaches.	Helps identify and maintain individual optimal arousal zones.	Personalized Wearable Devices
James–Lange Theory [35]	William James, Carl Lange	Physiological changes precede emotions.	Monitoring physiological changes can indicate emotional states.	Provides early indicators of emotional states.	Temperature Sensors
Cannon–Bard Theory [36]	Walter Cannon, Philip Bard	Physiological and emotional responses occur simultaneously.	Monitors both physiological and emotional states concurrently.	Provides a comprehensive understanding of simultaneous responses.	Simultaneous Physiological Monitoring
Schachter–Singer Theory [37]	Stanley Schachter, Jerome Singer	Emotions result from physiological arousal and cognitive interpretation.	Combines physiological data with contextual information.	Helps interpret and modulate emotional responses.	Multi-Modal Wearable Sensors

**Table 2:** Some wearable sensor applications in health monitoring and sports psychology [38].

Health Monitoring with Wearable Sensors in Sports Psychology	Body Part	Brand	Application
EEG monitoring for expanded focus and emotional control	Head	Muse™	Cognitive function, emotional control
Advanced EEG analysis, paired with Muse™ headband	Head	Opti Brain™	Cognitive function
Monitors emotional health	Wrist	Sentio Solutions	Emotional health
Head impact sensor, measures and transmits data on head forces	Head	CSx	Concussion monitoring
		X2 Biosystems	
Integrates EEG monitoring	Eyes	Smith Optics	Focus and emotional control
Advanced oxygenation monitoring	Forehead	Artinis Medical Systems	Oxygenation monitoring
Combines EEG and near-infrared sensors for cognitive function	Head	Neuroelectrics	Cognitive function exploration
Monitors activity, sleep, HR, HRV, temperature, and stress levels	Wrist	FitBit	Commercial, personal health
Monitors ECG, HR, HRV, RR, SpO_2_, and body temperature	Chest/Torso	Zephyr	Medical, research
		Carré Technologies	Medical, research
		Polar H10	Fitness, health monitoring
Posture correction	Lower back	Lumo Bodytech	Health and wellness
Monitors HR and temperature	Finger	OURA	Sleep tracking, fitness
Assesses cognitive function, especially after concussions	N/A	King-Devick	Cognitive assessment, sports
	N/A	HitCheck	
Focuses on attention and memory	N/A	BrainCheck	Cognitive assessment, sports
Monitors emotional health	N/A	T2	Emotional health

**Table 3:** Three-dimensional printing techniques for health monitoring with wearable sensors in sports psychology.

3D Printing Techniques	Materials	Description	Applications	Resolution and Accuracy	Wearable Sensors	Benefits (Athlete Psychology Evaluation)
Vat Photopolymerization (SLA, DLP)	Acrylate-based resins, stimulus-responsive materials.	Involves curing liquid photopolymer resin using a light source, layer by layer, to create detailed parts.	High precision, smooth surface finishes, medical research, complex optical components.	High resolution, accuracy due to photocurable material selection.	Glucose sensors [43,52], lactate sensors [43,52], sweat sensors [44], strain sensors [45,46,47], artificial skin [48], tactile sensors [48], oximeters [49], smart bandages, tattoo type sensors, EEG and ECG sensors [50].	Enables detailed psychological evaluations by measuring stress and fatigue levels through various biomarkers, contributing to a comprehensive understanding of mental states during performance.
Material Extrusion (FDM, FFF)	Thermoplastic elastomers, conductive inks, silicone elastomers, core–shell filaments.	Uses heated thermoplastic filament extruded through a nozzle to build objects layer by layer.	Prototyping, functional parts from thermoplastics, flexible material extrusion.	Contingent on material properties and process parameters; some studies emphasize functional integration, others high-fidelity printing.	Strain sensors [54,55], glucose sensors [56], lactate sensors [57], temperature sensors [58], electrocardiogram sensors [59,60,73].	Useful for real-time psychological stress analysis through physiological responses, allowing adjustments in training regimens to optimize psychological resilience.
Powder Bed Fusion (SLS, EBM)	Steel, metal powders.	SLS uses a laser to sinter powdered material, EBM uses an electron beam to melt metal powder in a vacuum.	High-strength, complex metal parts, aerospace and medical applications.	High resolution, accuracy, low error rates.	Force sensors [63].	Offers insights into the psychological impact of physical stress and fatigue during high-intensity activities, aiding in recovery and mental conditioning.
Material and Binder Jetting	Composite filaments, elastomers, functional inks, hydrogels.	Material jetting is similar to inkjet printing with photopolymer or wax; binder jetting uses a liquid binding agent on a powder bed.	Full-color prototypes, complex geometries, multi-material parts.	High precision crucial for functionality; intricate geometries, detailed features.	Glucose sensors [43,52], lactate sensors [43,52], sweat sensors [44], strain sensors [45,46,47], artificial skin [48], tactile sensors [48], oximeters [49], smart bandages, tattoo type sensors, EEG and ECG sensors [50].	Facilitates multi-dimensional psychological evaluations, tracking emotional stability and stress response through sensor data integration, offering a more nuanced approach to mental health management.
Multi-Jet Fusion (MJF)	Conductive graphene nanoplate–carbon nanotube (GC) ink, voxelated conductive elastomers.	Uses inkjet printheads to apply a fusing agent to powder, followed by heat to sinter material.	Functional parts with good mechanical properties.	High printing resolution, high dimensional accuracy.	Sensors with healthcare functionalities [66], components with varying electrical conductivities [67,68].	Enables complex psychological evaluations through advanced sensors capable of capturing minute physiological changes and emotional reactions under various performance and stress scenarios.
Directed Energy Deposition (DED)	Ceramics, polymers with piezoelectric properties (Pyzoflex^®^ sensors).	Uses focused thermal energy (laser, electron beam, plasma arc) to fuse materials as they are deposited.	New part creation or repair of existing components, high-value industries.	High resolution and accuracy in powder flow monitoring for DED processes.	Pyzoflex^®^ sensors [69].	Allows for the monitoring of psychological and physical responses in real time, particularly useful in assessing how athletes cope with stress and pressure during competitions and training.
Hybrid Additive Manufacturing (HAM)	Conductive polymer composites, novel conductive photo-resins.	Combines 3D printing with subtractive processes like CNC machining.	Greater precision and surface finish in complex parts.	Advanced precision and surface finish due to the integration of additive and subtractive techniques.	High-performance conductive polymer composites (CPCs), potentially wearable sensors [70,71].	Advances athlete psychological evaluation by seamlessly integrating sensors into sportswear, allowing for the continuous assessment of mental states without disrupting the athlete’s performance or comfort.
